# Correction: Janjetovic et al. Novel Vitamin D3 Hydroxymetabolites Require Involvement of the Vitamin D Receptor or Retinoic Acid-Related Orphan Receptors for Their Antifibrogenic Activities in Human Fibroblasts. *Cells* 2024, *13*, 239

**DOI:** 10.3390/cells15070624

**Published:** 2026-03-31

**Authors:** Zorica Janjetovic, Shariq Qayyum, Sivani B. Reddy, Ewa Podgorska, S. Gates Scott, Justyna Szpotan, Alisa A. Mobley, Wei Li, Vijay K. Boda, Senthilkumar Ravichandran, Robert C. Tuckey, Anton M. Jetten, Andrzej T. Slominski

**Affiliations:** 1 Department of Dermatology, University of Alabama at Birmingham, Birmingham, AL 35294, USA; 2 Brigham’s Women’s Hospital, Harvard University, Boston, MA 02115, USA; 3 College of Pharmacy, The University of Tennessee Health Science Center, Memphis, TN 38163, USAvb@uthsc.edu (V.K.B.); 4 School of Molecular Science, The University of Western Australia, Perth 6009, Australia; 5 Cell Biology Section, NIEHS, National Institutes of Health, Research Triangle Park, NC 27709, USA; jetten@niehs.nih.gov; 6 Cancer Chemoprevention Program, Comprehensive Cancer Center, University of Alabama at Birmingham, Birmingham, AL 35294, USA; 7 VA Medical Center, Birmingham, AL 35294, USA

## Figure Legend

In the original publication [1], there were some errors in the text that we discovered post factum. All of them were caused by the existence of multiple versions of the paper and the loss of the correct one. 

There was a mistake in the legend of Figure 4. The word “not” should be replaced by the word “partially”. The correct legend appears below.

**Figure 4.** Expression of CYP27B1 is partially required for the effects of 20(OH)D3 and 20,23(OH)_2_D3 on fibroblasts. (**A**) Pathways for the metabolism of vitamin D3 by CYP11A1 and CYP27B1. Enzymes are in red, derivatives are in blue and reactions are in green color. (**B**) WT (control) or si-CYP27B1 cells were incubated with 20(OH)D3 or 20,23(OH)_2_D3 (1, 10 or 100 nM) for 24 h, and proliferation was measured using the MTS assay. Data are expressed relative to control cells treated with the ethanol vehicle (% of control) and are means ± SD (*n* ≥ 6), * *p* < 0.05, ** *p* < 0.01, *** *p* < 0.001, by the *t*-test. (**C**) the supernatant was collected from WT or si-CYP27B1 cells treated with the secosteroids (100 nM) or the ethanol vehicle, as indicated, for 24 h. Total collagen content of samples was determined using the Sircol collagen assay. The data were analyzed using the *t*-test, with a *p* value of 0.05 or less indicating statistical significance. (**D**) Graphs were constructed to show the expression of the genes involved in fibrosis and inflammation in WT and si-CYP27B1 fibroblasts. Human dermal fibroblasts (WT or si-CYP27B1) were treated with 100 nM secosteroids, as shown, for 24 h. RNA was isolated and the expression of genes involved in fibrosis and inflammation was measured using RT-PCR. Data are presented as graphs showing fold-changes relative to the ethanol vehicle for each cell type.

## Text Correction

In the Abstract “either 20(OH)D3 or 20,23(OH)_2_D3, indicating that their actions are independent of 1α-hydroxylation.” should be changed to “20(OH)D3 and 20,23(OH)_2_D3 on cell proliferation but affected collagen synthesis.”; also, in the last sentence of the Abstract “not required” should be changed to “required only for anti-fibrogenic activity”. A correction has been made to Abstract:

**Abstract:** We investigated multiple signaling pathways activated by CYP11A1-derived vitamin D3 hydroxymetabolites in human skin fibroblasts by assessing the actions of these molecules on their cognate receptors and by investigating the role of CYP27B1 in their biological activities. The actions of 20(OH)D3, 20,23(OH)_2_D3, 1,20(OH)_2_D3 and 1,20,23(OH)_3_D3 were compared to those of classical 1,25(OH)_2_D3. This was undertaken using wild type (WT) fibroblasts, as well as cells with *VDR*, *RORs*, or CYP27B1 genes knocked down with siRNA. Vitamin D3 hydroxymetabolites had an inhibitory effect on the proliferation of WT cells, but this effect was abrogated in cells with silenced *VDR* or *RORs*. The collagen expression by WT cells was reduced upon secosteroid treatment. This effect was reversed in cells where VDR or RORs were knocked down where the inhibition of collagen production and the expression of anti-fibrotic genes in response to the hydroxymetabolites was abrogated, along with ablation of their anti-inflammatory action. The knockdown of *CYP27B*1 did not change the effect of 20(OH)D3 and 20,23(OH)_2_D3 on cell proliferation but affected collagen synthesis. In conclusion, the expression of the VDR and/or RORα/γ receptors in fibroblasts is necessary for the inhibition of both the proliferation and fibrogenic activity of hydroxymetabolites of vitamin D3, while CYP27B1 is required only for anti-fibrogenic activity.

In Section 3.4, paragraph two, “regardless of” should be changed to “depending on”, “or” should be changed to “but affects” and “that these secosteroids do not require” should be changed to “indicating a complex role of”. A correction has been made to Section 3.4, paragraph two:

As expected, a strong, concentration-dependent inhibitory effect on proliferation was observed in control cells treated with either 20(OH)D3 or 20,23(OH)_2_D3, when compared to the vehicle control (Figure 4B). This effect was also seen in si-CYP27B1 fibroblasts, and there were no significant differences in the degree of inhibition between control and si-CYP27B1 cells at any of the concentrations tested. Both secosteroids reduced the amount of soluble collagen present in the cell supernatants, depending on whether *CYP27B1* was expressed or not (*p* < 0.05) (Figure 4C). These results indicate that the expression of CYP27B1 is not necessary for the action of 20(OH)D3 or 20,23(OH)_2_D3 on proliferation but affects collagen synthesis in human fibroblasts and indicating a complex role of 1α-hydroxylation for their biological activity. 

In Section 4, paragraph four, “also retained” should be changed to “lost”; “anti-proliferative” should be added before “actions” and “but is necessary for anti-fibrotic activities.” should be added before “Similar”. A correction has been made to Section 4, paragraph four:

CYP27B1 can hydroxylate CYP11A1-derived vitamin D3 hydroxymetabolites at C1α (Figure 4A), modifying their biological activity through an increased affinity for the VDR [18,55]. For example, 20(OH)D3 lacks calcemic activity whereas 1,20(OH)_2_D3 does display moderate calcemic activity, although this is lower than for 1,25(OH)_2_D3 [25]. Therefore, whether 20(OH)D3 and 20,23(OH)_2_D3 act directly on the VDR (or RORs) to cause their biological effects or whether they must first undergo 1α-hydroxylation is an important question. To explore whether the antifibrotic activities of 20(OH)D3 and 20,23(OH)_2_D3 derivatives are dependent on hydroxylation at the C1α position, we silenced the *CYP27B1* gene in human fibroblasts. Proliferation, as well as collagen synthesis and the expression of *COL1A1* and COL3A1 genes, were inhibited in the control cells (WT) by these two secosteroids. The two secosteroids lost the ability to downregulate the expression of *TGFB1* and *THBS1* genes in si-CYP27B1 fibroblasts. Thus, we conclude that the C1α-hydroxylation of 20(OH)D3 and 20,23(OH)_2_D2 by CYP27B1 to produce1,20(OH)_2_D3 and 1,20,23(OH)_3_D3, respectively, is not required for their anti-proliferative actions on fibroblasts but is necessary for anti-fibrotic activities. Similar results have been reported for 20(OH)D2 in keratinocytes, where the silencing of the *CYP27B1* gene did not prevent this secosteroid from the stimulation of the keratinocyte differentiation program [23]. 

In Section 5, “biological” should be changed to “anti-proliferative”, “not” should be removed and “these” should be changed to “anti-fibrotic”. A correction has been made to Section 5:

The current study validates that the novel CYP11A1-derived vitamin D3 hydroxymetabolites have anti-fibrotic activities in human fibroblasts, supporting previous reports for murine fibroblasts in vitro and in vivo [17,55]. These effects are similar to those of 1,25(OH)_2_D3 and hydroxyl-pregnacalciferols (derivatives with a shortened side chain) [54]. Thus, non-calcemic vitamin D hydroxyderivatives are excellent candidates for treatment of local or systemic fibrosing diseases. In regard to the underlying mechanisms of the action of CYP11A1-derived vitamin D3 hydroxyderivatives on human fibroblasts in reducing fibrosis and inflammation, the present study demonstrates that such activities in human fibroblasts depend on the intact VDR, as well as on RORα and RORγ. In addition, although CYP27B1 might be important for the metabolism of these and other vitamin D3 derivatives in some settings, its presence is not required for the observed anti-proliferative actions of 20(OH)D3 and 20,23(OH)_2_D3 on fibroblasts. This is an important finding, since in the classical vitamin D activation pathway, the precursor molecule 25(OH)D3 requires hydroxylation by CYP27B1 to generate biologically active 1,25(OH)_2_D3 [3,85]. The possibility that novel vitamin D3 hydroxymetabolites can also exert antifibrotic activities through action on additional nuclear receptors represents a future challenge. Such activity would be context-dependent and dependent on the intracellular ligand concentration. In summary, this study identifies VDR or RORα/γ receptors as promising targets for antifibrogenic and anti-inflammatory activities by CYP11A1-derived hydroxyderivatives in human fibroblasts, with CYP27B1 being required for anti-fibrotic effects. 

The authors state that the scientific conclusions are unaffected. This correction was approved by the Academic Editor. The original publication has also been updated.
